# To Brake or Not to Brake? Personality Traits Predict Decision-Making in an Accident Situation

**DOI:** 10.3389/fpsyg.2019.00134

**Published:** 2019-02-05

**Authors:** Uijong Ju, June Kang, Christian Wallraven

**Affiliations:** ^1^Department of Brain and Cognitive Engineering, Cognitive Systems Lab, Korea University, Seoul, South Korea; ^2^Department of Biomedical Science, Interdisciplinary Affective Neuroscience Lab, Korea University, Seoul, South Korea; ^3^Empathy Research Institute, Goyang, South Korea

**Keywords:** virtual reality, decision-making, accident situation, personality, psychopathy, driving

## Abstract

Many situations require decisions to be made in very little time—in emergency or accident situations such decisions will carry potentially harmful consequences. Can we predict how people react in such situations from their personality traits alone? Since experimental tests of accident situations are not possible in the real world, existing studies usually employ text-based surveys or post-situation assessments, making predictions and generalization difficult. In the present study, we used virtual reality to create a more life-like situation in order to study decision-making under controlled circumstances. In our experiment, participants trained in an immersive car simulation to complete a race-course as fast as possible. In the testing phase, pedestrians appeared on the course without warning, forcing participants to react. The experiment used a one-shot design to avoid pre-meditation and to test naïve, rapid decision-making. Participants' reactions could be classified into two categories: people who tried to brake, and people who potentially endangered pedestrians by not braking or conducting hazardous evasion maneuvers. Importantly, this latter group of participants scored significantly higher on psychopathy-related traits among a set of personality-related factors. Additional personality factors, as well as age, gender, gaming expertise, and driving experience did not significantly influence participants' decision-making. This result was true for both a Korean sample (*N* = 94) and an independently-tested German sample (*N* = 94), indicating cross-cultural stability of the results. Overall, our results demonstrate that decision-making in an extreme, simulated accident situation is critically influenced by personality traits.

## Introduction

Often, one person's decision decides the fate of many people. This can both have negative outcomes as in a recent fatal plane crash in France that was initiated by a pilot, as well as positive outcomes as in a continued broadcasting of warnings during the 2011 tsunami in Japan that cost two government workers their lives but saved many other peoples' lives. Even though some of these decisions are clearly pre-meditated, many difficult decisions have to be made in very little time and can happen anytime even in every-day life: for example, a person driving fast with their car, as they are late for an important interview when, suddenly, a pedestrian appears on the road. In such a situation, several driving actions (from risky maneuvering around the pedestrian to full emergency braking) are possible, which are potentially triggered by trying to avoid hurting the pedestrian. What aspects affect decision-making in such situations? Is it possible to predict from a personality profile how a person would react in an extreme, dangerous situation like this?

According to the 2-systems model of social behavior, behaviors and actions are the consequences of reflective and impulsive processes (Strack and Deutsch, 2004). For the former, people perceive situational cues and deliberately decide their behavior based on their knowledge or experiential values. In contrast, for the latter, it is posited that people perceive situational cues and then an automatic association network activates a behavior with the strongest links. Importantly, this associative network can be learned and can also be formed by reflective processes (Johnson and Hirst, 1993). Personality is intertwined with these two processes as the explicit and implicit self-concepts that arise from a life-time of experiences (Strack and Deutsch, 2004; Back et al., 2009; Mcadams and Olson, 2010). This means that in an extreme situation, some people may take a risk based on explicit or implicit self-concepts derived from life-time experiences related to general situations. Since both experiences and acquired concepts are different across individuals, it follows that personality affects reflective and impulsive processing and consequently influences decision-making. Although much research has shown connections between explicit, self-reported concepts and life outcomes in general (i.e., Vazire and Gosling, 2004), comparatively less is known about the connection of explicit personality traits and observable behavior with several previous studies finding clear discrepancies between self-reported traits and observed actions (West and Jan Brown, 1975; Todd et al., 2007; Tassy et al., 2013b).

If one wants to study decision-making in extreme situations, it is clearly not possible to submit participants to accident situations in real-life. Computer-based experiments and in particular virtual reality (VR) represent a solution to this problem, as they provide a realistic and immersive, yet “safe” testing bed. As one example, in the context of research on fire evacuation, VR has been shown to provide a safe and controllable environment for investigating behaviors in critical situations compared to other research methods (Kinateder et al., 2014). A few studies have started to investigate decision-making in driving situations using VR: two car driving simulation studies, for example, found that people with more traffic violations in the real world drove their virtual car faster (Schwebel et al., 2006) and braked less heavily (Stephens and Groeger, 2009). Except for some anxiety traits, however, no other personality traits were significantly correlated with virtual driving behavior (Stephens and Groeger, 2009) nor were these able to predict risky driving in VR (Schwebel et al., 2006). In the present study, we seek to further investigate the connection between personality traits and decision-making in an accident situation using VR.

Importantly, none of the VR studies on simulated driving have so far clearly linked personality traits with decision-making of the participants, leaving the question open of how decisions in a realistic, potentially dangerous situation may be influenced by personality traits. One of the limitations of the mentioned VR studies was that participants were usually able to predict that something would happen next: for example, in the car driving study, similar traffic situations were replicated many times (Schwebel et al., 2006; Stephens and Groeger, 2009), leading participants to pay particular attention to these situations therefore potentially biasing or changing their decision-making criteria. In the present study, we avoided making participants aware of the upcoming decision-event, thereby trying to determine how much their *impulsive* decision-making (that is, decisions without explicit instructions) would be predictable based on their personality traits.

In particular, we designed a car simulation in which participants first had to train to finish a course under difficult driving conditions. After the training, participants then were told that their next lap would be the final testing lap in which we recorded their driving skills. During this lap and after a sharp curve, pedestrians suddenly appeared on a bridge, frantically waving and shouting “Stop.” Since the pedestrians blocked the full extent of the road, participants had no choice but to steer the car off the bridge, to brake, or to try and steer the car past the pedestrians, thereby potentially endangering them. In this situation, we were looking for potential influences of personality traits on that final decision.

Our selection of potential personality traits to is motivated from a research context in which associations between decision-making and personality were investigated in “extreme” situations. For example, in the well-known “trolley dilemma” or in the “footbridge dilemma,” participants are asked to decide whether to sacrifice one person in order to save five people from an incoming train (Foot, 1967; Thomson, 1985). If participants agree to sacrifice one person to save the other five, this decision is often called a utilitarian judgment (Mill, 1863; Mill and Crisp, 1998), whereas if they do not agree to sacrifice the one person, the decision is called a deontological judgment (Greene et al., 2001, 2004; Cushman et al., 2006; Valdesolo and Desteno, 2006; Hauser et al., 2007; Mikhail, 2007). When investigating what kinds of people make which type of decision, interestingly, in this type of dilemma the utilitarian trait is positively related to psychopathy in many cases (Glenn et al., 2010; Bartels and Pizarro, 2011; Koenigs et al., 2012; Wiech et al., 2013; Kahane et al., 2015) and negatively correlated with empathic concern (Crockett et al., 2010; Choe and Min, 2011; Kahane et al., 2015), which also highlights a link between decision and certain personality traits. Based on these studies, we therefore chose psychopathy as well as empathy-related traits as potential personality factors.

It has been shown that psychopathy is not only related to moral dilemma decision-making, but also associated with risky decision-making in different situations. For example, in the balloon analog risk task, a high level of psychopathy predicts risky behavior and secondary psychopathy showed correlation with risky decision-making in young adults (Hunt et al., 2005). Additionally, psychopathy showed correlations with self-reported irresponsible and criminal risk-taking compared to other external disorders for jailed inmates (Swogger et al., 2010). Such correlations may be explained by associations of high psychopathy with low moral identity (Glenn et al., 2010), lack of violence inhibition (Blair, 1995), or lack of ability to integrate rule and stimulus values (Baskin-Sommers et al., 2016). Hence, a high level of psychopathy may lead one to ignore basic moral codes in extreme situations.

Additionally, a lack of empathy is considered a critical component of psychopathy (Cleckley, 1964), which means that empathy itself also may influence decision-making in such situations. Several studies support a relationship between empathy and the ethical decision-making process (Mencl and May, 2009) and decision-making in medical emergencies (Loewenstein, 2005). Such correlations can be derived from empathy's two main components: the cognitive understanding of another person's state and the experiencing of the other's affective state (Hoffman, 1984). Hence, in the context of our present study, empathy may impact whether or not one is willing to risk potential harm to others in extreme circumstances, such as accident situations.

Although the goal of our study was to determine the influence of personality on decision-making, it may be possible that other factors would determine the decision of the participants. For example, a previous, inter-cultural driving behavior study found that drivers in the U.S, Spain, Germany, and Brazil seem to have differences in risk perception (Sivak et al., 1989). To investigate whether such differences also extend to intuitive decision-making in our task, we included both a Korean sample and a German sample in our study, testing how well the results of one sample would generalize to the other. Additional factors that may play a role in the decision-making were also tested for their influence in our study, including gender (Fumagalli et al., 2010), the amount of driving experience (Lajunen and Summala, 1995), computer gaming experience (Anderson et al., 2010), as well as general subjective experience during the simulation (Chou and Ting, 2003).

In short, in the present study we investigated decision-making in an emergency situation. Based on our experimental paradigm and the present state of research as outline above, we hypothesize that in (1) this condition, participants' responses can be divided into several categories based on their control input to the car and that (2) these different decision categories can in part be predicted based on particular personality traits.

## Materials and Methods

### Game Design

**Figure 1:**
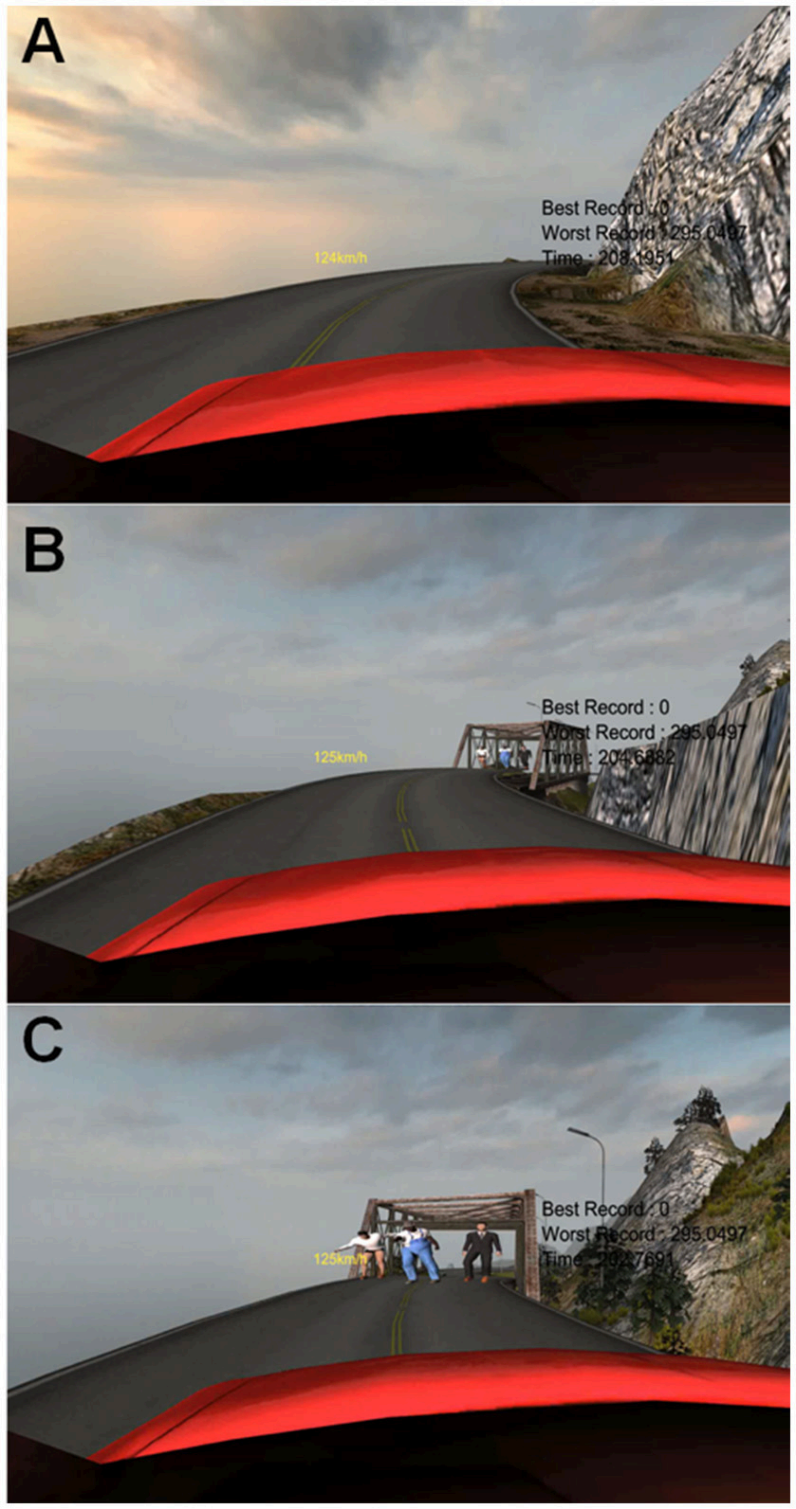
Screenshots of the accident situation. **(A)** Right before the event. **(B)** Three pedestrians suddenly appeared on the road. **(C)** The middle person shouting “stop!”. Note that the pedestrians block the whole width of the road with rocks to the right side preventing a potential evasive maneuver.

We used Unity3D 4.6.1f1 (Unity Technologies, San Francisco, USA; a game engine tool also used for virtual reality development) together with open source assets to design our car-driving simulation (the assets we used were based on sources found at https://www.assetstore.unity3d.com/en/#!/content/10). Figure 1 shows more details of the setup and the visuals. In order to implement realistic car controls, we added a wheel-pedal interface (Joystick, Power Racer 270 DX; Seoul, KR), which allowed two degrees of freedom and used pedals for acceleration and braking. In order to make controls easy to learn, only forward/backward accelerator and left-right steering were used in the experiment. Visualization was done using a state-of-the-art commercial head mounted display (HMD, Oculus Rift DK2; Irvine, USA; resolution = 800 × 600 px at 60 frames per second) which afforded stereoscopic depth and also allowed us to precisely track and represent yaw, pitch, and roll movements from a gyroscope, accelerometer and magnetometer inside the headset. Participants were able to move their head and look around in the car and the virtual environment if they wished. Engine and environmental sounds were provided through ear-phones to further increase immersion in the virtual reality environment.

We used a two-group, quasi-experimental design for the experiment. Participants had to complete three training sessions and one test session. During the training session, they learned how to control the car as well as the layout of the race course in virtual reality. The aim of the training was to finish the course in <5 min. In order to provide further motivation and enhance concentration on the task, the current speed of the car, as well as the best and worst lap times for the course were presented on screen. The training was automatically finished if participants reached the finish line or if 5 min had passed. If a participant did not reach the goal of 5 min within three training sessions, they were excluded from the experiment—in our case, none of the participants failed to reach this goal.

Before entering the test session, participants were told that the lap time in the following run was going to be their final time and that they should concentrate to make a fast lap. During the test lap, as soon as the car turned a specific corner of the course, an accident situation occurred: three people appeared on a bridge and started walking toward the car. Since the brake was set to low sensitivity during the whole game, it was not possible to easily stop the car. In addition, the gaps between people were too small for the car to pass through. As soon as the car was close to the first person, the person in the middle shouted “stop” and raised their hands to indicate stopping. The rightmost person froze as if in fear, whereas the left character also moved their hands to indicate stopping. The experiment was automatically finished when participants either collided with a virtual character or tried to steer the car off the cliff followed by a crash on the ground (see Figure 1 and Video S1). During this whole event, participants' control inputs to the accelerator (hit or not), brake (hit or not), and steering wheel (left/right) were record in each frame, starting at the time when participants turned a corner until the experiment terminated with the collision or the drop.

Since VR setups can sometimes result in uncomfortable experiences, if a participant felt dizzy or had any other symptoms of virtual-reality sickness, they were told to immediately take off the HMD and to notify the experimenter.

### Sample Size and Participants

The required sample size was calculated through an a priori power analysis (G^*^ power, Faul et al., 2007) based on a two-tailed *t*-test with non-equal allocation ratio. In independent pilot experiments, we found that participants' decisions in the event could be group into two categories: those people who tried to avoid hitting the pedestrians by hitting the brake, and those people who endangered participants by trying to squeeze past them or by even not braking at all (see Supplementary Material for a discussion and analysis of the experimental data with three instead of two groups). We termed these two groups *Don't Ignore* and *Ignore*. Additionally, the pilot showed that decision-making in our task had a group allocation ratio for the two decision categories *Don't Ignore*/*Ignore* of 2. We then determined the sample size using an effect size of *d* = 0.8 for the personality traits at a standard power of 1-beta = 80%. As the analysis included six different personality scales, we used a Bonferroni-corrected alpha-value of alpha = 0.05/6. The resulting sample size was 90 participants in total with 60 participants in the *Don't Ignore* group and 30 in the *Ignore* group.

We then recruited participants until that sample size was achieved in our Korean sample and tested for the consistency of the results in an independent sample in Germany. A total of 203 participants were recruited for our experiment [103 from Korea, 100 from Germany, 89 males, 114 females, between 20 and 39 years of age and with a mean age of 25.3 years (SD = 4.27)]. Korean participants were Korea University students, and German participants were recruited from the student population of Tübingen using online advertisement. Fifteen participants felt 3D-sickness symptoms such as dizziness during the training session and voluntarily ended the experiment (9 Korean, 6 German, 9 males, 6 females), leaving a total of 188 participants for analysis [94 from Korea (47 males, 47 females), 94 from Germany (33 males, 61 females)]. All participants had normal or corrected-to normal vision and did not report any neurological disease, nor were they currently under psychological treatment or medication.

### Game Experience and Expertise

To assess subjective game experience, we used the Game Experience Questionnaire [GEQ, developed by K. Poels, W. A. Ijsselsteijn, and Y. A. W de Kort at the Game Experience Lab Eindhoven (NL), (http://www.gamexplab.nl) in the European project FUGA]. Additionally, we queried participants' experience with driving video games [citing examples, such as Grand Theft Auto (Rockstar North Co., Ltd., Edinburgh, Scotland)]. We also asked whether participant possessed a driving license and how they would judge their driving skills in general. Finally, we asked them to describe their action or decision in the accident situation, as well as why they chose to act as they did.

### Personality Questionnaires

To measure personality traits, we used Levenson's self-report psychopathy scale (Levenson et al., 1995), the balanced emotional empathy scale (Mehrabian, 1996), and parts of the interpersonal reactivity index (Davis, 1983), including fantasy scale, empathic concern, personal distress, and perspective taking. All personality scales were normalized between 0 and 100% for comparison. We used Korean versions of the psychopathy scale (Lee and Js, 2007), balanced emotional empathy scale (Chung, 2012) and inter personal reactivity index (Kang et al., 2009) validated for Korean participants and the corresponding original English versions for the German participants. As for the latter, the experimenter offered to clarify any issues with the English terms if necessary—no participant reported any problems in understanding, however.

### Procedure

Before the experiment, the experimenter explained the aim of the game, how to control the car, the procedure of the study, and helped participants to fit the equipment for comfortable use. After the setup procedure, the experimenter started the training sessions three times. After each training session, the experimenter checked on participants, answered any questions and asked whether they would like to take a short break. After the training sessions, the experimenter reminded participants that the following lap would be the test session and that their lap time would be recorded. Right after the accident situation, the simulation finished and participants filled in the personality questionnaires, the Game Experience Questionnaire (GEQ), and additional experiment-related questions.

### Ethics

The study was approved by the local ethics committee at Korea University (1040548-KU-IRB-15-125-A-2) and carried out in accordance with the relevant guidelines and regulations. Written informed consent was obtained from all participants. Since we hid the actual goal of the experiment and presented an unexpected accident situation to the participants that they were not able to fully avert, we made sure to minimize potential emotional impact on participants: first, the game was ended before the actual collision happened and we gave neither visual nor sound effects of collision. Second, directly after the accident situation, the experimenter explained the purpose of the experiment and enquired whether participants felt any (potentially longer-lasting) negative experience from the event. Even though many participants said that they were surprised and even shocked by the event, all participants felt comfortable after hearing our explanation and talking about their reactions.

## Results

### Decision-Making in the Accident Situation

All participants successfully completed three trial runs in the experimental setup before moving on to the test round, in which—supposedly—their final lap time would be measured. During this final course, after participants had exited a sharp curve, pedestrians suddenly appeared on the road, blocking the path of the car. The simulation had a rather low brake sensitivity, and the course deliberately used an easy layout, leading participants to drive their car fast, so that they would not be able to come to a complete standstill by braking after seeing the pedestrians on the road. Since it was not possible to pass the pedestrians with the car (see Figure 1C), the experiment automatically finished after people either collided with a pedestrian or steered the car off the cliff (only one person did so). On average, the time between the appearance of the virtual pedestrians in view and the end of the event was 1.81 s (SD = 0.08 s).

As a first step, we analyzed the control behavior in the accident situation, which led to the creation of two decision categories for both participant samples: the *Don't ignore* group [participants tried to avoid the situation by stopping to hit accelerator and hitting the brake, or by using the steering wheel more than accelerator—the Supplementary Material (see Supplementary Tables 4–7) also includes an analysis with this latter group subdivided further] and the *Ignore* group (participants made no use of the brake, or the amount of accelerator use was larger than the amount of steering wheel use). In addition, we checked how participants described their behavior in the situation in the debriefing questionnaire. Table 1 shows participants' self-description of their decision together with our group assignment, which is fully consistent for 95.7% of all participants. In the following analyses, inconsistent results were excluded—results for all participants differed only marginally and can be found in the Supplementary Material.

**Table 1:** Group-assignment (based on control behavior) and self-descriptions.

**Group-assignment based on control behavior**	**Participants' self-descriptions**	**Number of participants (Korean/German)**
Don't ignore	Tried to pass the people, but failed	15 (11/4)
	Hit the brake	42 (23/19)
	Tried to hit the brake, but too late to stop	61 (24/37)
	Went ahead	4 (2/2)
	I drove right and fell down with the car	1 (0/1)
	Take foot off the accelerator	2 (2/0)
	Turn off handle, but failed to avoid	1 (0/1)
		Total: 126 (62/64)
Ignore	Gave up to drive	3 (2/1)
	Ignored the people, since my goal was to finish the course	41 (22/19)
	Curious what happens after collision	6 (3/3)
	Hit the brake	3 (2/1)
	I panicked and forgot to brake	5 (3/2)
	Tried to pass the people, but failed	1 (0/1)
	Keep driving	3 (0/3)
		Total: 62 (32/30)

*Potential mismatches between the assignment and the self-description included five people in the Don't Ignore group, who described that they ignored the virtual people, four people in the Ignore group, who said that they had hit the brake, and another person, who claimed to have avoided the pedestrians. Analyses were done both on all 188 participants and on the subset of 180 (fully consistent) participants*.

The first important result from the analysis of the decision categories (Table 1) shows that about a third of all participants fell into the *Ignore* group, potentially endangering the participants (62 out of 186 participants, 32 in the Korean sample, 30 in the German sample). Second, the number of people in the two decision categories in both Korean and German samples was the same: a χ^2^ -test showed no significant differences (χ^2^ = 0.096, *p* = 0.756, this and all following statistical tests were implemented in SPSS, IBM, New York, USA).

### Control Input

Control input for both Korean and German samples was also similar as determined by a one-way MANOVA with the factor of culture (accelerator use: *F*_(1)_ = 2.719, η^2^ = 5361.787, *p* = 0.101; brake use: *F*_(1)_ = 1.126, η^2^ = 1460.511, *p* = 0.290; wheel use: *F*_(1)_ = 0.165, η^2^ = 188, *p* = 0.685). This shows that both groups reacted similarly in the event in terms of control input amount.

### Personality Profiles

To compare personality profiles between the two decision categories, first, we used two sample *t*-tests (corrected for differences in sample size and for multiple comparisons using Bonferroni correction with α = 0.05/6) to find potential differences across the six personality scales (see Table 3, Supplementary Table 1). For the Korean sample, the *Ignore* group had significantly higher psychopathy [*t*_(88)_ = 3.9, *p* < 0.001, *Hedges' g* = 0.87] and lower perspective taking [*t*_(88)_ = 3.41, *p* = 0.001, *Hedges' g* = 0.75]. The strongest difference for psychopathy between the groups was confirmed for the German sample, for which the *Ignore* group also showed higher psychopathy [*t*_(88)_ = 3.68, *p* < 0.001, *Hedges' g* = 0.84], as well as lower empathic concern [*t*_(88)_ = 3.04, *p* = 0.003, *Hedges' g* = 0.69] compared to the *Don't ignore* group (a two- and three-factor analysis of the psychopathy subscales is discussed in the Supplementary Materials).

Additionally, when analyzing both samples together in a logistic regression with factors cultural background, personality traits and other additional factors, we found significant effects of psychopathy and perspective taking on decision-making—at the same time there were no significant effects of cultural background and actual decision-making (see Supplementary Table 3), confirming that variability *within* a culture was larger than variability *across* cultures.

### Influence of Other Factors

Next, we investigated potential differences between the two decision categories in terms of gender, driving license possession, amount of experience with video games, and age for both the Korean and German samples (see Table 2): χ^2^-tests showed no significant differences in terms of gender (Korean: χ^2^ = 0.2, *p* = 0.655, German: χ^2^ = 3.112, *p* = 0.078), driving license possession (Korean: χ^2^ = 2.977, *p* = 0.084, German: χ^2^ = 0.383, *p* = 0.536) or amount of experience with video games (Korean: χ^2^ = 0.358, *p* = 0.549, German: χ^2^ = 1.500, *p* = 0.221) between the two groups. Two sample *t*-tests also showed no significant differences in age between the two decision categories (Korean: *t*_(88)_ = 1.72, *p* = 0.088, *Hedges' g* = 0.39, German: *t*_(88)_ = 1.68, *p* = 0.096, *Hedges' g* = 0.38). A further analysis also yielded no significant differences between the two decision categories concerning participants' subjective game experience for both the Korean and the German sample (see Supplementary Table 2). Hence, these factors had little to no influence on the decision-making in the accident situation.

**Table 2:** Statistics for the two groups.

		**Don't ignore**	**Ignore**
Korean	Total number	62	32
	Accelerator hit (SD)	47.56 (36.76)	111.47 (26.59)
	Brake hit (SD)	33.00 (38.09)	0
	Wheel hit (SD)	80.61 (39.00)	79.66 (32.69)
	Group assignment (Fully consistent/Potential Mismatch)	60/2	30/2
	Gender–Male/female (all)	29/31 (30/32)	16/14 (17/15)
	Driving license–Yes/No (all)	43/17 (44/18)	16/14 (17/15)
	Violent video game experience–Yes/No (all)	26/34 (27/35)	15/15 (16/16)
	Age	24.03 (1.91)	23.15 (2.56)
German	Total number	64	30
	Accelerator hit (SD)	38.23 (35.25)	102.17 (22.35)
	Brake hit (SD)	40.15 (39.22)	0
	Wheel hit (SD)	79.81 (30.52)	75.03 (30.48)
	Group assignment (Fully consistent/Potential Mismatch)	62/2	28/2
	Gender–Male/Female (all)	19/43 (19/45)	14/14 (14/16)
	Driving license–Yes/No (all)	54/8 (56/8)	23/5 (25/5)
	Violent video game experience–Yes/No all)	29/33 (30/34)	17/11 (17/13)
	Age (SD)	27.31 (4.95)	25.63 (5.05)

*The “Group assignment” row lists the number of people for which self-descriptions were fully consistent with control behavior and the number of people for which there was a potential mismatch (see Table 1)*.

**Table 3:** Differences in personality scales between the two groups.

	**Personality scales**	**Don't ignore**	**Ignore**	**Effect size**	**95% C.I of the difference**	
					**Lower**	**Upper**
Korean	Psychopathy	31.0 ± 0.9	36.9 ± 1.3	0.872^*^	2.9	9.0
	Balanced empathy	67.6 ± 1.4	63.7 ± 1.8	0.376	−8.6	0.7
	Fantasy scale	66.0 ± 2.1	58.5 ± 3.8	0.415	−15.7	0.5
	Empathic concern	65.3 ± 1.6	62.0 ± 2.6	0.249	−9.1	2.6
	Personal distress	49.8 ± 2.5	50.1 ± 3.9	0.015	−8.6	9.2
	Perspective taking	68.8 ± 1.4	58.9 ± 3.2	0.751^*^	−15.7	−4.0
German	Psychopathy	29.2 ± 1.2	37.9 ± 2.2	0.836^*^	4.0	13.4
	Balanced empathy	67.7 ± 1.7	59.5 ± 3.3	0.556	−14.8	−1.5
	Fantasy scale	61.5 ± 2.0	59.7 ± 3.4	0.109	−9.4	5.8
	Empathic concern	68.7 ± 1.9	56.1 ± 4.5	0.692^*^	−20.8	−4.4
	Personal distress	43.4 ± 1.9	41.3 ± 3.3	0.131	−9.4	5.2
	Perspective taking	66.2 ± 1.9	61.5 ± 2.8	0.312	−11.5	2.1

*Values are re-scaled to 0–100% for all scales for easier comparison. Numbers are given as mean ± SEM*.

* *indicates p < 0.05 as determined by corrected two sample t-tests*.

## Discussion

The present study has for the first time found reliable relationships between impulsive decision-making in a realistic, difficult situation, and personality traits. In two independently-tested samples from two different cultural backgrounds, decision-making of the participants could be divided into two categories, which confirms our first hypothesis. This division was consistent in both participants' control input (use of accelerator) and in their self-report of how they reacted in the event (giving specific reasons for their reactions). Importantly, we showed that psychopathic tendencies were significantly related to the decision outcome in this accident situation, which confirms our second hypothesis.

### Influence of Personality on Decision-Making

Overall, our findings fit well into a behavior-based decision-making model suggesting personality as one of the four factors affecting impulsive decision-making processes (Sinclair and Ashkanasy, 2005). Other studies have shown, for example, that “extreme intuitive” groups needed a lower number of clues to solve puzzles and had consistently different patterns of personality (Westcott, 1968), or that performance on implicit learning tasks was affected by personality differences related to intuition or perceiving (Woolhouse and Bayne, 2000). Additionally, decision-making in high neuroticism personalities was found to be impaired during high pressure conditions but not during low pressure conditions (Byrne et al., 2015). A recent study on sports players (Otten, 2009) also found links between personality traits and performance under pressure (sport confidence and self-confidence which in turn affect perceived control and focus abilities). The present study extends these results to another realistic, high-pressure situation in which participants are expected to decide quickly and for which we found decision-making to be significantly affected by personality measures with psychopathic traits having a crucial impact.

Previous studies have found that high psychopathy is associated with low moral identity (Glenn et al., 2010): although people with higher psychopathy know which actions are morally right, they do not seem concerned about the consequences of their decisions (Cima et al., 2010) and often judge harming others acceptable in order to achieve a valuable outcome (Koenigs et al., 2012). In addition, psychopathic traits do not only change moral judgment but also moral action (Tassy et al., 2013a), which is evidenced by a high level of moral permissibility for harming other people in emergency situations (Young et al., 2012). When looking at the self-descriptions of the decision-making in the accident situation in our data, many participants in the *Ignore* group describe their behavior based on the original goal (i.e., trying to reach the goal fast), whereas self-descriptions in the *Don't ignore* group seem more based on common rules in society (i.e., trying to avoid the accident). This difference in descriptions also fits with the observed differences in personality traits. This may imply that people in the *Ignore* group would tend to think that it is permissible to pursue the original goal of the experiment with the (virtual) persons being obstacles in reaching this goal. In contrast, participants in the *Don't ignore* group may try to avoid to harm the pedestrians—even though they are in a virtual environment and their lap time will be affected by their decision.

An alternative explanation for the observed differences between groups may be attentional effects related to competition between the manifest task (that is, the explicitly informed goal—getting a fast lap time) and the latent task (that is, the hidden goal—avoiding the pedestrians). Previous studies found that high-psychopathic people have attentional deficits that lead them to ignore irrelevant or hidden information (Hiatt et al., 2004; Newman et al., 2010). Based on the description of the participants obtained in the questionnaires, however, all participants were aware of the situation and reported to have had ample time to react. Additionally, four participants in the *Ignore* group described that they were curious as to what “would happen after the collision.”

### Influence of Other Factors on Decision-Making

Importantly for the generalizability of our results, however, other factors—including cultural background, gender, driving experience, gaming expertise as well as subject gaming experience—did not differ significantly between the two decision categories. This means that the influence of these factors on the final decision—while not fully excludable—at best is low and certainly much weaker than the influence of the personality factors.

Previous research has shown differences in driving behavior between real-world roads and racing-game roads for highly-experienced drivers vs. participants without driving experience (Ciceri and Ruscio, 2014). Although overall the German participant group tended to be more experienced in terms of driver license possession, the two decision categories showed no differences in driving experience (see Supplementary Results). Future studies looking for specific differences along these lines should compare a larger breadth of drivers from novice to high experience to investigate potential differences in decision-making.

It has been shown that cultural differences for moral judgments occur as people from a Western, Educated, Industrialized, Rich, and Democratic (“WEIRD”) (Rozin, 2010) background emphasize individual rights more, whereas non-“WEIRD” cultures consider community and duty to be more important (Haidt et al., 1993; Graham et al., 2011; Buchtel et al., 2015). For example, helping other people in-group is considered as a duty by Indians whereas Americans consider it as a personal choice (Miller, 1997)—a difference that was explained by Westerners focusing more on the outcome and individual rights compared to Eastern participants, who focus more on their own social duties (Graham et al., 2016). The decision in the present experiment, however, does not directly involve a conflict between individual rights and general duty, but rather between the experimenter's requirement and general ethical values (see the discussion above on self-reports). Furthermore, the amount of time afforded for the decision was much lower than in the moral dilemma experiments, which may have further diminished any cultural differences—indeed, it would be an interesting study topic to investigate in more detail the time at which said differences may emerge in decision-making.

We also found neither differences for participant gender in terms of decision-making, nor strong personality differences *per se* between genders (see Supplementary Results). This is in line with previous studies that claimed gender differences in moral orientation to be relatively small and restricted to specific decision categories [female participants preferred care-based judgments, whereas male participants preferred justice-based judgment (Jaffee and Hyde, 2000)]. Importantly, the ratios of men and women in the two decision categories were also not significantly different, which indicates similar decision strategies across genders.

Previous studies have found that increased levels of exposure to violent video games may affect empathy and prosocial behavior (Anderson et al., 2010). However, in our study, more than half of the people had no experience with driving video games, which made it difficult to determine detailed correlations. We did find that people who played such games before had significantly lower personal distress (see Supplementary Results)—a result that could be related to the hypothesized desensitization effects of (violent) video games (Anderson et al., 2010), although this issue remains contentious (Ferguson, 2007).

### Potential Limitations of Present Study

An important issue arising from any VR study, however, is the fact that people may still see this “as a game,” knowing that colliding with the virtual people would not result in real damage. Many participants, however, did react with surprise and shouts to the sudden appearance of pedestrians, suggesting that our scenario carried some realism [see also the VR-replications of Milgram's obedience experiment and the bystander-effect that found similar behavior to the original versions (Slater et al., 2006, 2013)]. One way to test this aspect more explicitly, would be to run another set of participants with non-human obstacles on the road, in which case the differences between the groups would be predicted to be smaller.

A similar issue related to the “game aspect” is the overall quality of presence in the simulation. A lack of presence could have influenced the decision-making as well, as participants may not have felt that they were part of the simulation. Results from the post-game experience questionnaire we conducted (see Supplementary Results), however, include specific dimensions of “immersion” and “flow,” two components associated with presence (Nacke and Lindley, 2008). Both of the rating values were high enough (see published results by Poels et al. on the GEQ) to imply that our experiment setup provided sufficient presence to the participants.

Further, specific technical aspects that may affect the presence and perceived realism of our simulation are discussed in the following: First, the interface did not include detailed, realistic haptic feedback for the driving wheel and pedal beyond the default resistance of the device. Although no participant complained about the lack of realism in this regard, additional results from Francis et al. (2017) may point to a limited impact: in that study, the trolley dilemma was tested with people having to push an actual mannequin of a person, instead of a joystick interface, but decision ratios were not changed between the two conditions. Second, although we did match the movements of the in-game wheel to the interface wheel, enhanced immersion, and embodiment may result from a more realistic steering wheel visualization including a virtual body. In order to fully realize this, however, it will be necessary to implement real-time hand-tracking on the steering wheel as a generic, non-customized hand visualization may also break realism if appearance or motion do not match the participant's expectations. Finally, the visual appearance of the pedestrians could also have affected the decision-making as participants may have opted to “drive over” the pedestrians when they felt that they did not like their appearance. Since the time for a close-up evaluation of the character appearance was rather limited and one of the characters also shouted “Stop!” during the event, this may have led participants to react more realistically in this situation. Future studies, however, should employ additional questionnaire items to gauge the impact of the perceived effects of the game realism on participants' decisions in more detail beyond the GEQ dimensions.

Another potential limitation of our study is that we did not gather information about other parameterizations of personality: previous research on car driving behavior, for example, has tried to find correlations between previous accident experiences on the one hand and self-reported personality traits, driving tendency, as well as driving skills on the other hand. These studies suggested that sensation seeking, risk-taking, driving anger, and anxiety are related to accident incidences (Jonah, 1997; Deffenbacher et al., 2000; Oltedal and Rundmo, 2006). The Big Five—another well-known set of personality trait measures—has also been tested in association with decision-making: for example, extraversion showed correlations with traffic violation (Renner and Anderle, 2000) and fatal car accidents (Kirkcaldy and Furnham, 2000), neuroticism showed correlation with prevalence of car accidents (Lajunen, 2001) and aggressive driving (Matthews et al., 1991), whereas conscientiousness showed negative correlation with motor vehicle crashes (Winfred and Dennis, 2001) and agreeableness had an inverse relationship with both alcohol and non–alcohol related traffic violations (Brown et al., 2016). Although some previous research has investigated the Big Five in risk-taking contexts as mentioned above, in this study we chose to focus on psychopathy and empathic traits as these allow for a more nuanced and focused analysis of decision-making in extreme situations, and as these traits are also closer to clinical applications and interventions for treatment purposes (Hare and Neumann, 2008; Skodol et al., 2011). Although this may represent a statistical issue as the number of scales increases, future studies could add additional personality factors such as sensation seeking, impulsivity, and the Big Five.

### Future Work

Interestingly, our overall results are also in line with a recent study on autonomic vehicle behavior, which showed that participants favor to sacrifice pedestrians to save themselves at any cost (Bonnefon et al., 2016). Indeed, although our game offered the possibility to sacrifice oneself by driving off the cliff in order to avoid hitting the pedestrians, this option was chosen by only one participant. In this context, it is also important to note that the overall task was set as “racing” and people may not have expected that pedestrians could suddenly appear on the road. The rationale behind choosing this particular setup was that we wanted to make an unavoidable situation (it was impossible to pass the pedestrians) in which participants still had enough time to react. Other types of situations (such as pedestrians suddenly crossing the street, for example) would need to be tested in future research in order to generalize across a wider range of decision-making contexts.

Overall, our study for the first time provides evidence for the importance of psychopathic personality traits in determining peoples' decision in (simulated) accident situations in which fast reactions are required.

## Data Availability Statement

All data for the two samples can be accessed at the following link: https://drive.google.com/open?id=1Dq_OeGea1ZRD6DmuwxqDl0tzxkq7WISZ

## Author Contributions

JK and CW suggested the initial experiment idea. UJ designed the experiments, collected, and analyzed the data. CW helped with and supervised experiment design and analysis. UJ, JK, and CW wrote the paper.

### Conflict of Interest Statement

The authors declare that the research was conducted in the absence of any commercial or financial relationships that could be construed as a potential conflict of interest.

## References

[B1] Anderson C. A. Shibuya A. Ihori N. Swing E. L. Bushman B. J. Sakamoto A.. (2010). Violent video game effects on aggression, empathy, and prosocial behavior in eastern and western countries: a meta-analytic review. Psychol. Bull. 136, 151–173. 10.1037/a0018251 20192553

[B2] Back M. D. Schmukle S. C. Egloff B. (2009). Predicting actual behavior from the explicit and implicit self-concept of personality. J. Pers. Soc. Psychol. 97, 533–548. 10.1037/a0016229 19686006

[B3] Bartels D. M. Pizarro D. A. (2011). The mismeasure of morals: antisocial personality traits predict utilitarian responses to moral dilemmas. Cognition 121, 154–161. 10.1016/j.cognition.2011.05.010 21757191

[B4] Baskin-Sommers A. Stuppy-Sullivan A. M. Buckholtz J. W. (2016). Psychopathic individuals exhibit but do not avoid regret during counterfactual decision making. Proc. Natl. Acad Sci. U.S.A. 113, 14438–43. 10.1073/pnas.1609985113 27911790 PMC5167137

[B5] Blair R. J. R. (1995). A cognitive developmental-approach to morality - investigating the psychopath. Cognition 57, 1–29. 10.1016/0010-0277(95)00676-P 7587017

[B6] Bonnefon J. F. Shariff A. Rahwan I. (2016). The social dilemma of autonomous vehicles. Science 352, 1573–1576. 10.1126/science.aaf2654 27339987

[B7] Brown T. G. Ouimet M. C. Eldeb M. Tremblay J. Vingilis E. Nadeau L.. (2016). Personality, executive control, and neurobiological characteristics associated with different forms of risky driving. PLoS ONE 11:e150227. 10.1371/journal.pone.0150227 26910345 PMC4766103

[B8] Buchtel E. E. Guan Y. J. Peng Q. Su Y. J. Sang B. Chen S. X. (2015). Immorality east and west: are immoral behaviors especially harmful, or especially uncivilized? Pers. Soc. Psychol. Bull. 41, 1382–1394. 10.1177/0146167215595606 26253486

[B9] Byrne K. A. Silasi-Mansat C. D. Worthy D. A. (2015). Who chokes under pressure? The Big Five personality traits and decision-making under pressure. Pers. Indiv. Differ. 74, 22–28. 10.1016/j.paid.2014.10.009 28373740 PMC5376094

[B10] Choe S. Y. Min K. H. (2011). Who makes utilitarian judgments? The influences of emotions on utilitarian judgments. Judg. Decis. Mak. 6, 580–592. Available online at: http://journal.sjdm.org/11/11904/jdm11904.pdf

[B11] Chou T. J. Ting C. C. (2003). The role of flow experience in cyber-game addiction. Cyberpsychol. Behavior 6, 663–675. 10.1089/109493103322725469 14756934

[B12] Chung M.-S. (2012). A validation study of the korean-balanced emotional empathy scale. Korean J. Counsel. 13, 1781–1797. 10.15703/kjc.13.4.201208.1781

[B13] Ciceri M. R. Ruscio D. (2014). Does driving experience in video games count? Hazard anticipation and visual exploration of male gamers as function of driving experience. Transport. Res. Part F 22, 76–85. 10.1016/j.trf.2013.11.001

[B14] Cima M. Tonnaer F. Hauser M. D. (2010). Psychopaths know right from wrong but don't care. Social Cognitive and Affective Neuroscience 5, 59–67. 10.1093/scan/nsp051 20053752 PMC2840845

[B15] Cleckley H. M. (1964). The Mask of Sanity, an Attempt to Clarify Some Issues About the So-Called Psychopathic Personality. Saint Louis, MO: C. V. Mosby Co.

[B16] Crockett M. J. Clark L. Hauser M. D. Robbins T. W. (2010). Serotonin selectively influences moral judgment and behavior through effects on harm aversion. Proc. Natl. Acad Sci. U.S.A. 107, 17433–17438. 10.1073/pnas.1009396107 20876101 PMC2951447

[B17] Cushman F. Young L. Hauser M. (2006). The role of conscious reasoning and intuition in moral judgment: testing three principles of harm. Psychol. Sci. 17, 1082–1089. 10.1111/j.1467-9280.2006.01834.x 17201791

[B18] Davis M. H. (1983). Measuring individual-differences in empathy - evidence for a multidimensional approach. J. Pers. Soc. Psychol. 44, 113–126. 10.1037/0022-3514.44.1.113

[B19] Deffenbacher J. L. Huff M. E. Lynch R. S. Oetting E. R. Salvatore N. F. (2000). Characteristics and treatment of high-anger drivers. J. Counsel. Psychol. 47, 5–17. 10.1037/0022-0167.47.1.5

[B20] Faul F. Erdfelder E. Lang A. G. Buchner A. (2007). G^*^Power 3: a flexible statistical power analysis program for the social, behavioral, and biomedical sciences. Behav. Res. Methods 39, 175–191. 10.3758/BF03193146 17695343

[B21] Ferguson C. J. (2007). The good, the bad and the ugly: a meta-analytic review of positive and negative effects of violent video games. Psychiatric Q. 78, 309–316. 10.1007/s11126-007-9056-9 17914672

[B22] Foot P. (1967). The problem of abortion and the doctrine of double effect. Oxford Rev. 5, 5–15.

[B23] Francis K. B. Terbeck S. Briazu R. A. Haines A. Gummerum M. Ganis G.. (2017). Simulating moral actions: an investigation of personal force in virtual moral dilemmas. Sci. Rep. 7:9. 10.1038/s41598-017-13909-9 29066760 PMC5654774

[B24] Fumagalli M. Ferrucci R. Mameli F. Marceglia S. Mrakic-Sposta S. Zago S.. (2010). Gender-related differences in moral judgments. Cogn. Proc. 11, 219–226. 10.1007/s10339-009-0335-2 19727878

[B25] Glenn A. L. Koleva S. Iyer R. Graham J. Ditto P. H. (2010). Moral identity in psychopathy. Judg. Dec. Mak. 5, 497–505. Available online at: http://journal.sjdm.org/10/10316/jdm10316.pdf

[B26] Graham J. Meindl P. Beall E. Johnson K. M. Zhang L. (2016). Cultural differences in moral judgment and behavior, across and within societies. Curr. Opin. Psychol. 8, 125–130. 10.1016/j.copsyc.2015.09.007 29506787

[B27] Graham J. Nosek B. A. Haidt J. Iyer R. Koleva S. Ditto P. H. (2011). Mapping the moral domain. J. Pers. Soc. Psychol. 101, 366–385. 10.1037/a0021847 21244182 PMC3116962

[B28] Greene J. D. Nystrom L. E. Engell A. D. Darley J. M. Cohen J. D. (2004). The neural bases of cognitive conflict and control in moral judgment. Neuron 44, 389–400. 10.1016/j.neuron.2004.09.027 15473975

[B29] Greene J. D. Sommerville R. B. Nystrom L. E. Darley J. M. Cohen J. D. (2001). An fMRI investigation of emotional engagement in moral judgment. Science 293, 2105–2108. 10.1126/science.1062872 11557895

[B30] Haidt J. Koller S. H. Dias M. G. (1993). Affect, culture, and morality, or is it wrong to eat your dog. J. Pers. Soc. Psychol. 65, 613–628. 10.1037/0022-3514.65.4.613 8229648

[B31] Hare R. D. Neumann C. S. (2008). Psychopathy as a clinical and empirical construct. Ann. Rev. Clin. Psychol. 4, 217–246. 10.1146/annurev.clinpsy.3.022806.091452 18370617

[B32] Hauser M. Cushman F. Young L. Kang-Xing Jin R. Mikhail J. (2007). A dissociation between moral judgments and justifications. Mind Lang. 22, 1–21. 10.1111/j.1468-0017.2006.00297.x

[B33] Hiatt K. D. Schmitt W. A. Newman J. P. (2004). Stroop tasks reveal abnormal selective attention among psychopathic offenders. Neuropsychology 18, 50–59. 10.1037/0894-4105.18.1.50 14744187

[B34] Hoffman M. L. (1984). Interaction of affect and cognition in empathy, in Emotions, Cognition, and Behavior, eds, Izard C. E. Kagan J. Zajonc R. B. (New York, NY: Cambridge University Press), 103–131.

[B35] Hunt M. K. Hopko D. R. Bare R. Lejuez C. W. Robinson E. V. (2005). Construct validity of the Balloon Analog Risk Task (BART)–Associations with psychopathy and impulsivity. Assessment 12, 416–428. 10.1177/1073191105278740 16244122

[B36] Jaffee S. Hyde J. S. (2000). Gender differences in moral orientation: a meta-analysis. Psychol. Bull. 126, 703–726. 10.1037/0033-2909.126.5.703 10989620

[B37] Johnson M. K. Hirst W. (1993). MEM: Memory subsystems as processes, in Theories of Memory, eds. Collins A. F. Gathercole S. E. Conway M. A. Morris P. E. (Hillsdale, NJ: Lawrence Erlbaum Associates, Inc), 241–286.

[B38] Jonah B. A. (1997). Sensation seeking and risky driving: a review and synthesis of the literature. Accid. Anal. Prev. 29, 651–665. 10.1016/S0001-4575(97)00017-1 9316713

[B39] Kahane G. Everett J. A. C. Earp B. D. Farias M. Savulescu J. (2015). ‘Utilitarian’ judgments in sacrificial moral dilemmas do not reflect impartial concern for the greater good. Cognition 134, 193–209. 10.1016/j.cognition.2014.10.005 25460392 PMC4259516

[B40] Kang I. Kee S. Kim S. E. Jeong B. Hwang J. H. Song J. E. (2009). Reliability and validity of the Korean-version of interpersonal reactivity index. J. Korean Neuropsyc. Assoc. 48, 352–358.

[B41] Kinateder M. Ronchi E. Nilsson D. Kobes M. Müller M. Pauli P. (2014). Virtual reality for fire evacuation research, in 2014 Federated Conference on Computer Science and Information Systems (Warsaw: IEEE), 313–321.

[B42] Kirkcaldy B. Furnham A. (2000). Positive affectivity, psychological well-being, accident- and traffic-deaths, and suicide: an international comparison. Studia Psychol. 42, 97–104.

[B43] Koenigs M. Kruepke M. Zeier J. Newman J. P. (2012). Utilitarian moral judgment in psychopathy. Soc. Cogn. Affect. Neurosci. 7, 708–714. 10.1093/scan/nsr048 21768207 PMC3427868

[B44] Lajunen T. (2001). Personality and accident liability: are extraversion, neuroticism and psychoticism related to traffic and occupational fatalities? Pers. Indiv. Differ. 31, 1365–1373. 10.1016/S0191-8869(00)00230-0

[B45] Lajunen T. Summala H. (1995). Driving experience, personality, and skill and safety-motive dimensions in drivers self-assessments. Pers. Indiv. Differ. 19, 307–318. 10.1016/0191-8869(95)00068-H

[B46] Lee S. J. Js G. (2007). Characteristic analysis of domestic homicidal behavior based on psychopathy. Korean Assoc. Police Sci. Rev. 13, 1–23.

[B47] Levenson M. R. Kiehl K. A. Fitzpatrick C. M. (1995). Assessing psychopathic attributes in a noninstitutionalized population. J. Pers. Soc. Psychol. 68, 151–158. 10.1037/0022-3514.68.1.151 7861311

[B48] Loewenstein G. (2005). Hot-cold empathy gaps and medical decision making. Health Psychol. 24, S49–S56. 10.1037/0278-6133.24.4.S49 16045419

[B49] Matthews G. Dorn L. Glendon A. I. (1991). Personality-correlates of driver stress. Pers. Indiv. Differ. 12, 535–549. 10.1016/0191-8869(91)90248-A

[B50] Mcadams D. P. Olson B. D. (2010). Personality development: continuity and change over the life course. Ann. Rev. Psychol. 61, 517–542. 10.1146/annurev.psych.093008.100507 19534589

[B51] Mehrabian A. (1996). Manual for the Balanced Emotional Empathy Scale. Los Angeles, CA.

[B52] Mencl J. May D. R. (2009). The effects of proximity and empathy on ethical decision-making: an exploratory investigation. J. Bus. Ethics 85, 201–226. 10.1007/s10551-008-9765-5

[B53] Mikhail J. (2007). Universal moral grammar: theory, evidence and the future. Trends Cogn. Sci. 11, 143–152. 10.1016/j.tics.2006.12.007 17329147

[B54] Mill J. S. (1863). Utilitarianism. London: Parker, son, and Bourn.

[B55] Mill J. S. Crisp R. (1998). Utilitarianism. Oxford; New York, NY: Oxford University Press.

[B56] Miller J. G. (1997). Cultural conceptions of duty: implications for motivation and morality, in Motivation and Culture, eds. Munro D. Schumaker J. F. Carr S. C. (New York, NY: Routledge), 178–192.

[B57] Nacke L. Lindley C. (2008). Flow and immersion in first-person shooters: measuring the player's gameplay experience, in Proceedings of the 2008 Conference on Future Play: Research, Play, Share (Toronto, ON: ACM), 81–88. 10.1145/1496984.1496998

[B58] Newman J. P. Curtin J. J. Bertsch J. D. Baskin-Sommers A. R. (2010). Attention moderates the fearlessness of psychopathic offenders. Biol. Psychiatry 67, 66–70. 10.1016/j.biopsych.2009.07.035 19793581 PMC2795048

[B59] Oltedal S. Rundmo T. (2006). The effects of personality and gender on risky driving behaviour and accident involvement. Safety Sci. 44, 621–628. 10.1016/j.ssci.2005.12.003

[B60] Otten M. (2009). Choking vs. clutch performance: a study of sport performance under pressure. J. Sport Exerc. Psychol. 31, 583–601. 10.1123/jsep.31.5.583 20016110

[B61] Renner W. Anderle F.-G. (2000). Venturesomeness and extraversion as correlates of juvenile drivers' traffic violations. Accid. Anal. Prevent. 32, 673–678. 10.1016/S0001-4575(99)00103-7 10908140

[B62] Rozin P. (2010). The weirdest people in the world are a harbinger of the future of the world. Behav. Brain Sci. 33:108. 10.1017/S0140525X10000312 20546650

[B63] Schwebel D. C. Severson J. Ball K. K. Rizzo M. (2006). Individual difference factors in risky driving: the roles of anger/hostility, conscientiousness, and sensation-seeking. Accid. Anal. Prev. 38, 801–810. 10.1016/j.aap.2006.02.004 16527223

[B64] Sinclair M. Ashkanasy N. M. (2005). Intuition - myth or a decision-making tool? Manag. Learn. 36, 353–370. 10.1177/1350507605055351

[B65] Sivak M. Soler J. Tränkle U. Spagnhol J. M. (1989). Cross-cultural differences in driver risk-perception. Accid. Anal. Prev. 21, 355–362. 10.1016/0001-4575(89)90026-2 2765078

[B66] Skodol A. E. Bender D. S. Oldham J. M. Clark L. A. Morey L. C. Verheul R.. (2011). Proposed changes in personality and personality disorder assessment and diagnosis for DSM-5 Part II: Clinical application. Pers. Disord. 2, 23–40. 10.1037/a0021892 22448688

[B67] Slater M. Antley A. Davison A. Swapp D. Guger C. Barker C.. (2006). A virtual reprise of the stanley milgram obedience experiments. PLoS ONE 1:e000039. 10.1371/journal.pone.0000039 17183667 PMC1762398

[B68] Slater M. Rovira A. Southern R. Swapp D. Zhang J. J. Campbell C.. (2013). Bystander responses to a violent incident in an immersive virtual environment. PLoS ONE 8:052766. 10.1371/journal.pone.0052766 23300991 PMC3534695

[B69] Stephens A. N. Groeger J. A. (2009). Situational specificity of trait influences on drivers' evaluations and driving behaviour. Trans. Res. Part F Traffic Psychol. Behav. 12, 29–39. 10.1016/j.trf.2008.06.005

[B70] Strack F. Deutsch R. (2004). Reflective and impulsive determinants of social behavior. Pers. Soc. Psychol. Rev. 8, 220–247. 10.1207/s15327957pspr0803_1 15454347

[B71] Swogger M. T. Walsh Z. Lejuez C. W. Kosson D. S. (2010). Psychopathy and risk taking among jailed inmates. Crim. Just. Behav. 37, 439–452. 10.1177/0093854810361617 20419073 PMC2856971

[B72] Tassy S. Deruelle C. Mancini J. Leistedt S. Wicker B. (2013a). High levels of psychopathic traits alters moral choice but not moral judgment. Front. Hum. Neurosci. 7:229 10.3389/fnhum.2013.00229 23761743 PMC3671176

[B73] Tassy S. Oullier O. Mancini J. Wicker B. (2013b). Discrepancies between judgment and choice of action in moral dilemmas. Front. Psychol. 4:250. 10.3389/fpsyg.2013.00250 23720645 PMC3655270

[B74] Thomson J. J. (1985). The trolley problem. Yale Law J. 94, 1395–1415. 10.2307/796133

[B75] Todd P. M. Penke L. Fasolo B. Lenton A. P. (2007). Different cognitive processes underlie human mate choices and mate preferences. Proc. Natl. Acad. Sci. U.S.A. 104, 15011–15016. 10.1073/pnas.0705290104 17827279 PMC1986604

[B76] Valdesolo P. Desteno D. (2006). Manipulations of emotional context shape moral judgment. Psychol. Sci. 17, 476–477. 10.1111/j.1467-9280.2006.01731.x 16771796

[B77] Vazire S. Gosling S. D. (2004). e-Perceptions: personality impressions based on personal websites. J. Pers. Soc. Psychol. 87, 123–132. 10.1037/0022-3514.87.1.123 15250797

[B78] West S. G. Jan Brown T. (1975). Physical attractiveness, the severity of the emergency and helping: a field experiment and interpersonal simulation. J. Exp. Soc. Psychol. 11, 531–538. 10.1016/0022-1031(75)90004-9

[B79] Westcott M. R. (1968). Toward a Contemporary Psychology of Intuition; a Historical, Theoretical, and Empirical Inquiry. New York, NY: Holt.

[B80] Wiech K. Kahane G. Shackel N. Farias M. Savulescu J. Tracey I. (2013). Cold or calculating? Reduced activity in the subgenual cingulate cortex reflects decreased emotional aversion to harming in counterintuitive utilitarian judgment. Cognition 126, 364–372. 10.1016/j.cognition.2012.11.002 23280149 PMC3629560

[B81] Winfred A. J. Dennis D. (2001). Predicting motor vehicle crash involvement from a personality measure and a driving knowledge test. J. Prev. Interv. Commun. 22, 35–42. 10.1080/10852350109511209

[B82] Woolhouse L. S. Bayne R. (2000). Personality and the use of intuition: individual differences in strategy and performance on an implicit learning task. Eur. J. Pers. 14, 157–169. 10.1002/(SICI)1099-0984(200003/04)14:2<157::AID-PER366>3.0.CO;2-L

[B83] Young L. Koenigs M. Kruepke M. Newman J. P. (2012). Psychopathy increases perceived moral permissibility of accidents. J. Abnormal Psychol. 121, 659–667. 10.1037/a0027489 22390288 PMC4603562

