# Supplementary material for: To Brake or Not to Brake? Personality Traits Predict Decision-Making in an Accident Situation
Source: Front Psychol. 2019 Feb 5;10:134. doi: 10.3389/fpsyg.2019.00134 (PMC6370639; doi:10.3389/fpsyg.2019.00134)
Supplement: Supplementary file 1 [file Table_1.DOCX]

Supplementary Material

To brake or not to brake? Personality traits predict decision-making in an accident situation

Uijong Ju^1^, June Kang^2,3^, Christian Wallraven^1*^

*** Correspondence:** Corresponding Author: wallraven@korea.ac.kr

# Supplementary Results

## Analysis including all participants

The following analyses repeat the main analyses of the paper with all 188 participants, including the 9 participants (=4.3%) whose self-report does not match their control input.

For the Korean sample, we found that the *Ignore* group had significantly higher psychopathy values (*t*(92) = 3.58, *p* = .001, *Hedges’ g* = 0.78) and lower perspective taking (*t*(92) = 3.69, *p* < .001, *Hedges’ g* = 0.80) compared to the *Don’t ignore* group. For the German sample, the *Ignore* group had higher psychopathy scores (*t*(92) = 3.20, *p* = .002, *Hedges’ g* = 0.71), lower empathic concern (*t*(92) = 2.88, *p* = .005, *Hedges’ g* = 0.64) compared to the *Don’t ignore* group. These results are in full agreement with the results reported in the main paper (see Supplementary Table 1 below).

Next, potential differences between the two decision categories in terms of gender, driving license possession and amount of experience with violent video games for both the Korean and German samples were explored (see Table 2): χ2-tests showed no significant differences in terms of gender (Korean: *χ2* = 0.190, *p* = .663, German: *χ2* = 2.585, *p* = .108), driving license possession (Korean: *χ2* = 2.950, *p* = .086, German: *χ2* = 0.298, *p* = .585) or amount of experience with violent video games (Korean: *χ2* = 0.354, *p* = .552, German: *χ2* = 0.783, *p* = .376) between the two groups. A further analysis also showed no significant differences between the two decision categories concerning their subjective game experience for both Korean and German sample (see Supplementary Table 2 below).

Finally, we include all eighteen factors together with all participants to estimate which factors affect decision-making. Logistic regression results showed that psychopathy, perspective taking showed significant affected decision-making. Again, these results remain the same compared to the main paper.

## Sub-scales of psychopathy

Here, we ran subscale analysis of psychopathy to investigate the influence of the different factors separately. First, based on Levenson’s original classification (Levenson et al., 1995), we compare primary and secondary psychopathy between groups. Results showed that for both the Korean and the German sample, the *Ignore* group had significantly higher primary psychopathy compare to the *Don’t ignore* group (Korean: t(92) = 3.52, p = .001, *Hedges’ g =* 0.77, German: t(92) = 3.60, p = .001, *Hedges’ g* = 0.80) with no significant differences in secondary psychopathy. In addition, we choose a three-factor structure of psychopathy (Brinkley et al., 2008) to compare the subscales between groups. Results showed that both the Korean and German sample, the *Ignore* group had significantly higher egocentricism (Korean: t (92) = 2.21, *p =* .030, *Hedges’ g* = 0.48, German: *t*(92) = 3.14 , *p =* .002, *Hedges’ g* = 0.70) and callous factor (Korean: *t* (92) = 3.13, *p =* .002, *Hedges’ g* = 0.68, German: *t*(64) = 3.51, *p =* .001, *Hedges’ g* = 0.78) compared to the *Don't ignore* group. We found no significant differences concerning antisocial features.

## Correlation of personality profile scales

Potential interdependencies of the six personality scales themselves were assessed using Spearman correlations (corrected for multiple comparisons): psychopathy was negatively correlated with balanced emotional empathy scale (r = -0.34, p <.001), empathic concern (*r* = -0.38, *p* < .001), and perspective taking (*r* = -0.34, *p* < .001). The balanced emotional empathy scale (BEES) was positively correlated with the fantasy scale (*r* = 0.52, *p* < .001), empathic concern (*r* = 0.67, *p* < .001) personal distress (*r* = 0.19, *p* = .008) and perspective taking (*r* = 0.25, *p <*.001), whereas the fantasy scale was positively correlated with empathic concern (*r* = 0.45, *p* < .001) and personal distress (*r* = 0.32, *p* < .001). Additionally, empathic concern was positively correlated with perspective taking (*r* = 0.30, *p* < .001). Overall, effect sizes were relatively small and the significant correlations highlighted no unusual interdependencies. Hence, each of the scales examines largely independent aspects of personality.

## Influence of other factors

Here, we report analyses concerning differences in subjective game experience during the car driving scenario for the two groups. For this, we used the Game Experience Questionnaire (GEQ) that measures several dimensions of subjective experience. As a first analysis, we ran two sample t-test (corrected for differences in sample size and multiple comparisons) to investigate whether the two groups would have different subjective experiences in the VR setup in the Korean and German sample (see Supplementary Table 2). None of the seven dimensions proved to be different for the two groups (and this result did not change when including the 9 participants in the analysis), showing that from a subjective point of view the decision outcome did not result from different game experiences.

Since fewer Koreans possess a driving license in the age group we tested, we put additional emphasis on driving experience in the German sample and asked the German participants how they would judge their driving skills in general. The self-reported driving confidence values were compared across the two decision groups, and we found no significant differences (*t*(79) = 1.26, *p =*.212), showing that driving expertise *per se* did not impact on the decision.

In addition, variability in the extent of braking behavior is potential dependent variable effects on the decision. Therefore, in German sample brake group, we record brake time after the event and try to correlate with personality and game experiences dimensions. We did not find significant correlation in all personality dimension and game experiences dimension which shows that extent of braking behavior is not a critical factor of decision.

## Three-group model

In addition to the two-group model discussed in the main text, we also tested a more fine-grained three-group model for the decisions. For this three-group model, the *Ignore* group remained the same, but the *Don’t Ignore* group was split further into a group who hit the brake during the event (*Brake*) or who tried to steer to avoid the pedestrians (*Avoid*).

For this setup of decision categories, we ran an ANOVA to check for differences in personality or game experiences depending on the decision category (see Supplementary Table 5). We found effect of psychopathy for both Korean (*F*(2) = 7.57, *η^2^* = 353.324, *p =*.001) and German samples (*F*(2) = 5.16, *η^2^* = 587.616, *p =*.008). Additionally, perspective taking (*F*(2) = 5.596, *η^2^* = 70.93, *p =*.005) was significant for the Korean sample only and empathic concern was significant for the German sample only (*F*(2) = 4.19, *η^2^* = 1396.614, *p =*.018). Next, we used post-hoc t-tests (Tukey’s HSD) to check for specific differences in measures between the three decision categories (see Supplementary Table 6). These results showed that the *Ignore* group had significantly higher psychopathy values compared to both *Brake* (*p =*.002) and *Avoid* group (*p =*.012) in the Korean sample, as well as higher values compared to both *Brake* group (*p* = .012) and *Avoid* group (*p* = .033) for the German sample. Additionally, for the Korean sample, the *Ignore* group had a lower perspective taking values compared to the *Brake* group (*p =*.005).

In addition, χ2-tests showed that decision proportions overall did not differ in gender (*p =*.525), video game experience (*p =* .273), but did so in driving license possession (*p =*.017) in the Korean sample. For the German sample, gender (*p =*.206), driving license possession (*p =*.213), and video game experience (*p =*.648) did not significantly differ between groups (see Supplementary Table 8). Additionally, we ran an ANOVA to check for differences in age on the decision category for both Korean (*F*(2) = 1.71, *η^2^* = 11.600, *p =* .186) and German samples (*F*(2) = 1.44, *η^2^* = 35.838, *p =*.243) and found no effect. The difference for the Korean sample is due to the fact that Koreans in general possess fewer driving licenses compared to Germany. When looking at this more closely, we can see that all people in the *Avoid* group possess a driving license, whereas the other two groups are more evenly distributed. Given the relatively low sample size for the three-group split and the fact that these effects are not visible for the German sample, however, further experiments would be necessary to confirm such effects.

For the three-group assignment, there were no significant differences across cultures for group count for decision-making (*χ2* =0.107*,* *p =*.948), no interaction between recorded behavior and cultural backgrounds (MANOVA: accelerator: *p =*.101, brake: *p =*.290, wheel: *p =*.685) as well as cultural background (Supplementary Table 7).

Finally, we included all eighteen factors together with all participants to estimate which factors affected on three group model decision-making. Logistic regression results showed that psychopathy significant affected the classification of *Ignore* for both groups and additionally, immersion and age showed significant effects for the *Avoid* and *Ignore* groups. Again, psychopathy is highlighted as the main effect similarly to the analysis in the main paper. Additionally, the results showed that younger participants seemed to ignored pedestrians more compared to the *Avoid* group.

Overall, these results confirm the findings discussed in the main text concerning the two-group model. We were not able to tease apart further characteristics in either personality or game experience (with the possible exception of driving license possession in the Korean sample and age as well potentially immersion) that would allow us to reliably categorize a person as making a braking or an avoiding decision in this situation. Further studies with different contexts that would penalize either braking (by a closely following car) or avoiding (by other people blocking the avoiding path) could be useful in creating situations that would require more difficult decisions to be made.

# Supplementary Tables

|  | Personality scales | Don't ignore | Ignore | Effect size | 95% C.I of the difference | |
| --- | --- | --- | --- | --- | --- | --- |
|  |  |  |  |  | Lower | Upper |
| Korean | Psychopathy | 36.4±1.2 | 31.1±0.9 | 0.71* | 2.4 | 8.4 |
|  | Balanced emotional empathy | 67.5±1.3 | 63.8±1.7 | 0.36 | -8.1 | 0.8 |
|  | Fantasy scale | 65.7±2.2 | 57.8±3.7 | 0.42 | -16.0 | 0.2 |
|  | Empathic concern | 65.6±1.6 | 62.4±2.4 | 0.24 | -8.7 | 2.4 |
|  | Personal distress | 50.6±2.5 | 50.1±3.7 | 0.03 | -9.2 | 8.1 |
|  | Perspective taking | 68.5±1.3 | 58.0±3.0 | 0.80* | -16.2 | -4.9 |
| German | Psychopathy | 29.1±1.2 | 36.6±2.3 | 0.70* | 2.9 | 12.2 |
|  | Balanced emotional empathy | 67.8±2.2 | 60.6±2.9 | 0.49 | -13.6 | -0.8 |
|  | Fantasy scale | 62.0±2.0 | 60.1±3.3 | 0.11 | -9.2 | 5.4 |
|  | Empathic concern | 68.9±1.9 | 57.3±4.3 | 0.64* | -19.6 | -3.6 |
|  | Personal distress | 44.1±1.9 | 41.1±3.1 | 0.19 | -10.2 | 4.0 |
|  | Perspective taking | 66.1±1.9 | 61.3±2.7 | 0.32 | -11.5 | 1.8 |

**Supplementary Table 1.** Differences in personality scales between the two groups for all participants (include inconsistent participants). Values are re-scaled to 0-100% for all scales for easier comparison. Values are given as mean ± SEM. * signifies corrected significance with p < .05 as determined by two-sample t-tests

|  | Game experiences | Don't ignore | Ignore | *P*-value | 95% C.I of the difference | |
| --- | --- | --- | --- | --- | --- | --- |
|  |  |  |  |  | Lower | Upper |
| Korean | Competence | 50.3±2.1 | 51.9±3.5 | .681 | -6.0 | 9.2 |
|  | Immersion | 55.6±2.3 | 56.3±3.4 | .866 | -7.4 | 8.8 |
|  | Flow | 60.6±2.0 | 58.2±2.7 | .481 | -9.2 | 4.3 |
|  | Tension | 42.7±1.9 | 40.7±2.6 | .551 | -8.6 | 4.6 |
|  | Challenge | 55.9±1.7 | 58.8±2.8 | .345 | -3.2 | 8.9 |
|  | Negative affect | 33.3±2.3 | 30.4±3.0 | .453 | -10.5 | 4.7 |
|  | Positive affect | 52.5±2.9 | 54.5±3.4 | .673 | -7.4 | 11.3 |
| German | Competence | 49.8±2.8 | 55.3±4.1 | .270 | -4.3 | 15.3 |
|  | Immersion | 45.0±2.7 | 42.5±3.8 | .597 | -11.8 | 6.8 |
|  | Flow | 59.0±2.5 | 57.5±4.3 | .752 | -10.8 | 7.8 |
|  | Tension | 33.0±2.2 | 25.8±2.7 | .054 | -14.5 | 0.1 |
|  | Challenge | 49.0±2.2 | 43.9±2.6 | .170 | -12.4 | 2.2 |
|  | Negative affect | 19.3±2.1 | 17.9±2.8 | .704 | -8.4 | 5.7 |
|  | Positive affect | 53.0±2.9 | 62.1±4.3 | .082 | -1.2 | 19.4 |

**Supplementary Table 2.** Differences in game experiences between the two groups. Values are re-scaled to 0-100% for all scales for easier comparison. Values are given as mean ± SEM. There were no significant differences

| Predictor | B | S.E | Wald | df | Sig. | Exp(B) | 95% C.I for Exp(B) | |
| --- | --- | --- | --- | --- | --- | --- | --- | --- |
|  |  |  |  |  |  |  | lower | Upper |
| Psychopathy | 0.077 | 0.025 | 9.701 | 1 | 0.002* | 1.08 | 1.029 | 1.134 |
| Balanced emotional empathy | 0.013 | 0.023 | 0.302 | 1 | 0.583 | 1.013 | 0.968 | 1.059 |
| Fantasy scale | -0.006 | 0.013 | 0.238 | 1 | 0.626 | 0.994 | 0.969 | 1.019 |
| Empathic concern | -0.006 | 0.017 | 0.119 | 1 | 0.73 | 0.994 | 0.962 | 1.027 |
| Personal distress | -0.007 | 0.011 | 0.411 | 1 | 0.522 | 0.993 | 0.971 | 1.015 |
| Perspective taking | -0.029 | 0.014 | 4.231 | 1 | 0.04* | 0.971 | 0.944 | 0.999 |
| Competence | 0.009 | 0.012 | 0.53 | 1 | 0.467 | 1.009 | 0.985 | 1.033 |
| Immersion | -0.024 | 0.014 | 2.865 | 1 | 0.091 | 0.977 | 0.95 | 1.004 |
| Flow | -0.002 | 0.013 | 0.017 | 1 | 0.895 | 0.998 | 0.973 | 1.024 |
| Tension | -0.019 | 0.016 | 1.355 | 1 | 0.244 | 0.982 | 0.951 | 1.013 |
| Challenge | 0.012 | 0.017 | 0.521 | 1 | 0.47 | 1.012 | 0.979 | 1.046 |
| Negative affect | 0.001 | 0.015 | 0.003 | 1 | 0.959 | 1.001 | 0.971 | 1.031 |
| Positive affect | 0.011 | 0.012 | 0.807 | 1 | 0.369 | 1.011 | 0.987 | 1.035 |
| Nationality | -0.15 | 0.498 | 0.091 | 1 | 0.763 | 0.861 | 0.324 | 2.285 |
| Sex | -0.406 | 0.459 | 0.781 | 1 | 0.377 | 0.666 | 0.271 | 1.639 |
| Violent video game experience | -0.229 | 0.43 | 0.282 | 1 | 0.595 | 0.796 | 0.342 | 1.85 |
| License | -0.566 | 0.461 | 1.504 | 1 | 0.22 | 0.568 | 0.23 | 1.403 |
| Age | -0.079 | 0.053 | 2.285 | 1 | 0.131 | 0.924 | 0.833 | 1.024 |

**Supplementary Table 3.** Results of logistic regression. The regression tested the effect of personality, game experiences, nationality, sex, video game experiences, license possession, age on decision-making of all participants. * signifies corrected significance with p <.05

| Group-assignment based on control behavior | Participants’ self-descriptions | Number of participants  (Korean / German) |
| --- | --- | --- |
| Avoid | Tried to pass the people, but failed | 14 (11/3) |
|  | Tried to hit the brake, but too late to stop | 13 (2/11) |
|  | Went ahead | 1(0/1) |
|  | Take foot off the accelerator | 2(2/0) |
|  | Turn off handle, but failed to avoid | 1(0/1) |
|  |  | Total: 31 (15 / 16) |
| Brake | Hit the brake | 41 (23/19) |
|  | Tried to hit the brake, but too late to stop | 49 (22/26) |
|  | Went ahead | 3 (2/1) |
|  | I drove right and fell down with the car | 1 (0/1) |
|  | Tried to pass the people, but failed | 1 (0/1) |
|  |  | Total: 95 (47 / 48) |
| Ignore | Gave up to drive | 3 (2 / 1) |
|  | Ignored the people, since my goal was to finish the course | 41 (22 / 19) |
|  | Curious what happens after collision | 6 (3 / 3) |
|  | Hit the brake | 3 (2 / 1) |
|  | I panicked and forgot to brake | 5 (3 / 2) |
|  | Tried to pass the people, but failed | 1 (0 / 1) |
|  | Keep driving | 3 (0 / 3) |
|  |  | Total: 62 (32 / 30) |

**Supplementary Table 4.** Description of the decision-making in the event situations in the three-group model. For this analysis, the *Don’t Ignore* group is further divided into an *Avoid* and a *Brake* group.

| Source | Variable | *df* | Mean square | *F* | *Sig.* |
| --- | --- | --- | --- | --- | --- |
| Korean | Psychopathy | 2 | 353.324 | 7.566 | 0.001* |
|  | Balanced emotional empathy | 2 | 849.996 | 1.418 | 0.248 |
|  | Fantasy scale | 2 | 42.350 | 1.743 | 0.181 |
|  | Empathic concern | 2 | 8.901 | 0.701 | 0.499 |
|  | Personal distress | 2 | 0.498 | 0.017 | 0.983 |
|  | Perspective taking | 2 | 70.934 | 5.596 | 0.005* |
|  | Competence | 2 | 290.873 | 1.002 | 0.371 |
|  | Immersion | 2 | 13.991 | 0.042 | 0.959 |
|  | Flow | 2 | 57.948 | 0.248 | 0.781 |
|  | Tension | 2 | 64.754 | 0.290 | 0.749 |
|  | Challenge | 2 | 83.566 | 0.446 | 0.642 |
|  | Negative affect | 2 | 537.316 | 1.879 | 0.159 |
|  | Positive affect | 2 | 39.798 | 0.088 | 0.915 |
| German | Psychopathy | 2 | 587.616 | 5.155 | 0.008* |
|  | Balanced emotional empathy | 2 | 531.808 | 2.458 | 0.091 |
|  | Fantasy scale | 2 | 53.618 | 0.189 | 0.828 |
|  | Empathic concern | 2 | 1396.614 | 4.189 | 0.018* |
|  | Personal distress | 2 | 97.832 | 0.372 | 0.69 |
|  | Perspective taking | 2 | 298.459 | 1.303 | 0.277 |
|  | Competence | 2 | 313.561 | 0.624 | 0.538 |
|  | Immersion | 2 | 331.224 | 0.741 | 0.479 |
|  | Flow | 2 | 37.151 | 0.082 | 0.921 |
|  | Tension | 2 | 625.555 | 2.265 | 0.11 |
|  | Challenge | 2 | 321.054 | 1.16 | 0.318 |
|  | Negative affect | 2 | 54.173 | 0.209 | 0.812 |
|  | Positive affect | 2 | 1473.112 | 2.739 | 0.07 |

**Supplementary Table 5.** Results of ANOVAs. ANOVAs testing the effect of decision-making on personality and subjective game experience with the three-group model (* signifies *p* < .05).

| . | Personality scales | Avoid | Brake | Ignore | *P*-value (Avoid and Brake) | *P*-value (Brake and Ignore) | *P*-value (Avoid and Ignore) |
| --- | --- | --- | --- | --- | --- | --- | --- |
| Korean | Psychopathy | 30.6±1.9 | 31.1±0.9 | 36.9±1.3 | .960 | .002* | .012* |
|  | Balanced emotional empathy | 67.1±3.1 | 67.8±1.5 | 63.7±1.8 | .977 | .230 | .556 |
|  | Fantasy scale | 67.1±3.3 | 65.6±2.7 | 58.5±3.8 | .959 | .225 | .295 |
|  | Empathic concern | 64.0±2.9 | 65.7±2.0 | 62.0±2.6 | .906 | .466 | .879 |
|  | Personal distress | 49.0±3.6 | 50.1±3.1 | 50.1±3.9 | .984 | 1.000 | .985 |
|  | Perspective taking | 68.3±2.6 | 69.0±1.6 | 58.9±3.2 | .986 | .005* | .069 |
|  | Competence | 55.5±3.6 | 48.6±2.5 | 51.9±3.5 | .370 | .691 | .785 |
|  | Immersion | 56.6±3.9 | 55.3±2.8 | 56.3±3.4 | .970 | .970 | .999 |
|  | Flow | 60.7±4.7 | 60.6±2.2 | 58.2±2.7 | 1.000 | .786 | .866 |
|  | Tension | 41.2±4.0 | 43.3±2.3 | 40.7±2.6 | .884 | .755 | .996 |
|  | Challenge | 56.0±2.8 | 55.8±2.0 | 58.8±2.8 | .999 | .636 | .801 |
|  | Negative affect | 40.1±5.0 | 31.1±2.5 | 30.4±3.0 | .182 | .986 | .175 |
|  | Positive affect | 52.4±6.1 | 52.5±3.3 | 54.5±3.4 | 1.000 | .918 | .949 |
| German | Psychopathy | 28.2±2.9 | 29.4±1.3 | 36.6±2.3 | .922 | .012* | .033* |
|  | Balanced emotional empathy | 67.2±3.9 | 68.0±1.8 | 60.6±2.9 | .978 | .083 | .588 |
|  | Fantasy scale | 60.7±3.4 | 62.4±2.5 | 60.1±3.3 | .934 | .826 | .993 |
|  | Empathic concern | 67.4±3.9 | 69.3±2.2 | 57.3±4.3 | .928 | .015* | .177 |
|  | Personal distress | 43.8±4.5 | 44.3±2.2 | 41.1±3.1 | .993 | .674 | .855 |
|  | Perspective taking | 68.5±4.2 | 65.3±2.1 | 61.3±2.7 | .745 | .492 | .277 |
|  | Competence | 49.0±6.4 | 50.1±3.0 | 55.3±4.1 | .983 | .582 | .635 |
|  | Immersion | 50.0±5.6 | 43.3±3.0 | 42.5±3.8 | .519 | .985 | .488 |
|  | Flow | 57.8±5.0 | 59.4±2.9 | 57.5±4.3 | .965 | .924 | .999 |
|  | Tension | 29.9±5.3 | 34.0±2.3 | 25.8±2.7 | .673 | .092 | .704 |
|  | Challenge | 51.3±4.1 | 48.2±2.6 | 43.9±2.6 | .792 | .512 | .325 |
|  | Negative affect | 17.4±4.5 | 19.9±2.3 | 17.9±2.8 | .861 | .860 | .995 |
|  | Positive affect | 60.7±6.0 | 50.4±3.2 | 62.1±4.3 | .282 | .084 | .979 |

**Supplementary Table 6.** Differences in personality scales and game experiences between the three groups. Values are re-scaled to 0-100% for all scales for easier comparison with SEM (**p* < .05; post-hoc t test)

| Avoid | Predictor | B | S.E | Wald | df | Sig. | Exp(B) | 95% C.I for Exp(B) | |
| --- | --- | --- | --- | --- | --- | --- | --- | --- | --- |
|  |  |  |  |  |  |  |  | lower | upper |
|  | Psychopathy | -0.095 | 0.035 | 7.525 | 1 | 0.006* | 0.909 | 0.849 | 0.973 |
|  | Balanced emotional empathy | -0.019 | 0.03 | 0.405 | 1 | 0.525 | 0.981 | 0.925 | 1.041 |
|  | Fantasy scale | 0.013 | 0.018 | 0.527 | 1 | 0.468 | 1.014 | 0.977 | 1.051 |
|  | Empathic concern | -0.001 | 0.024 | 0.001 | 1 | 0.973 | 0.999 | 0.954 | 1.046 |
|  | Personal distress | 0.007 | 0.016 | 0.203 | 1 | 0.652 | 1.007 | 0.976 | 1.04 |
|  | Perspective taking | 0.037 | 0.02 | 3.433 | 1 | 0.064 | 1.038 | 0.998 | 1.08 |
|  | Competence | -0.023 | 0.018 | 1.634 | 1 | 0.201 | 0.978 | 0.944 | 1.012 |
|  | Immersion | 0.043 | 0.019 | 4.8 | 1 | 0.028* | 1.044 | 1.005 | 1.084 |
|  | Flow | -0.01 | 0.018 | 0.288 | 1 | 0.591 | 0.99 | 0.955 | 1.027 |
|  | Tension | -0.01 | 0.022 | 0.237 | 1 | 0.626 | 0.99 | 0.949 | 1.032 |
|  | Challenge | 0.007 | 0.024 | 0.092 | 1 | 0.762 | 1.007 | 0.962 | 1.055 |
|  | Negative affect | 0.03 | 0.021 | 1.981 | 1 | 0.159 | 1.031 | 0.988 | 1.075 |
|  | Positive affect | -0.001 | 0.017 | 0.006 | 1 | 0.938 | 0.999 | 0.966 | 1.032 |
|  | Age | 0.145 | 0.073 | 3.901 | 1 | 0.048* | 1.156 | 1.001 | 1.335 |
|  | [Nationality=Germany] | 0.339 | 0.663 | 0.261 | 1 | 0.609 | 1.403 | 0.383 | 5.148 |
|  | [Nationality=Korea] | 0 | . | . | 0 | . | . | . | . |
|  | [Sex=F] | 0.444 | 0.654 | 0.461 | 1 | 0.497 | 1.559 | 0.433 | 5.619 |
|  | [Sex=M] | 0 | . | . | 0 | . | . | . | . |
|  | [Violent video game experience=O] | 0.598 | 0.604 | 0.979 | 1 | 0.322 | 1.818 | 0.557 | 5.936 |
|  | [Violent video game experience =X] | 0 | . | . | 0 | . | . | . | . |
|  | [License=O] | 0.828 | 0.696 | 1.415 | 1 | 0.234 | 2.29 | 0.585 | 8.965 |
|  | [License=X] | 0 | . | . | 0 | . | . | . | . |
| Brake | Psychopathy | -0.07 | 0.026 | 7.431 | 1 | 0.006* | 0.932 | 0.887 | 0.981 |
|  | Balanced emotional empathy | -0.01 | 0.024 | 0.185 | 1 | 0.667 | 0.99 | 0.944 | 1.038 |
|  | Fantasy scale | 0.004 | 0.013 | 0.077 | 1 | 0.781 | 1.004 | 0.978 | 1.031 |
|  | Empathic concern | 0.009 | 0.018 | 0.254 | 1 | 0.614 | 1.009 | 0.975 | 1.044 |
|  | Personal distress | 0.006 | 0.012 | 0.266 | 1 | 0.606 | 1.006 | 0.983 | 1.03 |
|  | Perspective taking | 0.027 | 0.015 | 3.288 | 1 | 0.07 | 1.027 | 0.998 | 1.057 |
|  | Competence | -0.004 | 0.013 | 0.115 | 1 | 0.735 | 0.996 | 0.971 | 1.021 |
|  | Immersion | 0.018 | 0.014 | 1.559 | 1 | 0.212 | 1.018 | 0.99 | 1.047 |
|  | Flow | 0.006 | 0.014 | 0.186 | 1 | 0.666 | 1.006 | 0.979 | 1.033 |
|  | Tension | 0.028 | 0.017 | 2.698 | 1 | 0.1 | 1.028 | 0.995 | 1.063 |
|  | Challenge | -0.019 | 0.018 | 1.126 | 1 | 0.289 | 0.981 | 0.948 | 1.016 |
|  | Negative affect | -0.01 | 0.016 | 0.41 | 1 | 0.522 | 0.99 | 0.958 | 1.022 |
|  | Positive affect | -0.013 | 0.012 | 1.147 | 1 | 0.284 | 0.987 | 0.963 | 1.011 |
|  | Age | 0.061 | 0.055 | 1.265 | 1 | 0.261 | 1.063 | 0.956 | 1.183 |
|  | [Nationality=Germany] | 0.054 | 0.521 | 0.011 | 1 | 0.918 | 1.055 | 0.38 | 2.93 |
|  | [Nationality=Korea] | 0 | . | . | 0 | . | . | . | . |
|  | [Sex=F] | 0.413 | 0.478 | 0.747 | 1 | 0.387 | 1.512 | 0.592 | 3.858 |
|  | [Sex=M] | 0 | . | . | 0 | . | . | . | . |
|  | [Violent video game experience=O] | 0.138 | 0.447 | 0.095 | 1 | 0.758 | 1.148 | 0.478 | 2.755 |
|  | [Violent video game experience =X] | 0 | . | . | 0 | . | . | . | . |
|  | [License=O] | 0.505 | 0.479 | 1.109 | 1 | 0.292 | 1.656 | 0.647 | 4.237 |
|  | [License=X] | 0 | . | . | 0 | . | . | . | . |

**Supplementary Table 7.** Results of logistic regression testing the effect of personality scales, game experiences, cultural background, age, sex, license possession, violent video game experiences on decision-making with the three-group model (baseline set to the *Ignore* condition).

|  |  | Avoid | Brake | Ignore |
| --- | --- | --- | --- | --- |
| Korean | Total number | 15 | 47 | 32 |
|  | Accelerator hit (SD) | 54.80 (36.39) | 45.26 (36.97) | 111.47 (26.59) |
|  | Brake hit (SD) | 0 | 43.53 (38.15) | 0 |
|  | Wheel hit (SD) | 107 (29.26) | 72.19 (38.18) | 79.66 (32.69) |
|  | Group assignment (Fully consistent/Potential Mismatch) | 15/0 | 45/2 | 30/2 |
|  | Gender–Male/Female  (include inconsistent) | 9/6 (9/6) | 20/25 (21/26) | 16/14 (17/15) |
|  | Driving license – Yes/No  (include inconsistent) | 14/1 (14/1) | 29/16 (30/17) | 16/14 (17/15) |
|  | Violent video game experience – Yes/No  (include inconsistent) | 9/6 (9/6) | 17/28 (18/29) | 15/15 (16/16) |
|  | Age (SD) | 24.53 (2.36) | 23.87 (2.77) | 23.16 (2.40) |
| German | Total number | 16 | 48 | 30 |
|  | Accelerator hit (SD) | 60.00 (29.74) | 30.98 (34.19) | 102.17 (22.35) |
|  | Brake hit (SD) | 0 | 53.54 (36.46) | 0 |
|  | Wheel hit (SD) | 92.50 (26.85) | 75.58 (30.75) | 75.03 (30.48) |
|  | Group assignment (Fully consistent/Potential Mismatch) | 15/1 | 47/1 | 28/2 |
|  | Gender–Male/Female  (include inconsistent) | 6/9 (6/10) | 13/34 (13/35) | 14/14 (14/16) |
|  | Driving license – Yes/No  (include inconsistent) | 11/4 (12/4) | 43/4 (44/4) | 23/5 (25/5) |
|  | Violent video game experience – Yes/No  (include inconsistent) | 6/9 (7/9) | 23/24 (23/25) | 17/11 (17/13) |
|  | Age (SD) | 28.12 (4.16) | 27.04 (5.20) | 25.63 (5.05) |
| **Supplementary Table 8.** Further information about each of the three groups. | | | | |

**References**

Brinkley, C.A., Diamond, P.M., Magaletta, P.R., and Heigel, C.P. (2008). Cross-Validation of Levenson's Psychopathy Scale in a Sample of Federal Female Inmates. *Assessment* 15**,** 464-482.

Levenson, M.R., Kiehl, K.A., and Fitzpatrick, C.M. (1995). Assessing Psychopathic Attributes in a Noninstitutionalized Population. *Journal of Personality and Social Psychology* 68**,** 151-158.
